# Comparing ecological relevance of climate velocity indices

**DOI:** 10.1038/s41598-025-32377-0

**Published:** 2026-02-13

**Authors:** Laure Moinat, Iaroslav Gaponenko, Stéphane Goyette, Jérôme Kasparian

**Affiliations:** 1 Institute for Environmental Sciences, University of Geneva, Bd Carl Vogt 66, 1211 Geneva 4, Switzerland; 2 Group of Applied Physics, University of Geneva, Rue de l’Ecole de médecine 20, 1211 Geneva 4, Switzerland; 3 DQMP, University of Geneva, Quai Ansermet 24, 1211 Geneva 4, Switzerland

**Keywords:** Climate sciences, Ecology, Ecology, Environmental sciences

## Abstract

Climate change is causing species ranges to shift. Population survival requires species to shift at a sufficient pace to accommodate with climate. However, estimating the velocity of climate change requires an assumption about its direction. This can be done via two alternative methods: the gradient-based methods and the recently introduced Monte-cArlo iTerative Convergence metHod (MATCH). In this work, we investigate how the rates of North-American birds and marine species range shifts correspond to the velocity of climate change (in the latitude, longitude and elevation/depth direction) calculated using either of these methods. These velocities are evaluated against the centroid of each considered species’ observed distribution range, based on the Audubon Christmas Bird Count and the NOAA Global Marine Data databases. We find that the MATCH method better describes the observed latitudinal and elevation/depth range shifts of the species, which is twice the Gradient performance. Developing robust method to evaluate the velocity of species could help in the planning of assisted migration.

## Introduction

Global climate change is already having a drastic impact on ecosystems, as well as individual species and organisms^1^. Besides adaptation via, e.g., evolutionary processes and the associated physiological and phenological changes^2^, life can respond to changing environments by shifting to locations with more adequate conditions^3^. Such shifts in distribution ranges are already observable in the environment for many species or biomes^4^. They result from a combination of individuals or groups willing to get closer to their ecological niche conditions, and of differential rates of birth, survival, and death in regions where climate becomes either more or less favorable. Therefore, the velocity (magnitude and direction) of the shift of distribution ranges is governed by the mobility of individuals, the generation time, as well as dispersal and differential reproduction and survival rates. As a result, the survival of species or biomes critically depends on their ability to shift sufficiently fast to keep pace with adequate environmental conditions.

To address this question it is necessary to translate the temporal and the spatial climate model outputs e.g., temperature, into a joint spatiotemporal information. To this end, the concept of the velocity of climate change^5,6^ has been introduced by Loarie et al.^5^, who proposed a *velocity index* to estimate the spatial velocity *v* of climate change. It is defined as the ratio of the local rate of change (temporal derivative, termed “temporal gradient” by these authors) of a scalar field $\psi$ (representing *e*.*g*., temperature, or, alternatively, precipitation, *etc*.) to the magnitude of the spatial gradient of $\psi$ (denoted $||\psi ||$):

1 $$\begin{aligned} v = \frac{\partial \psi }{||\nabla \psi || \partial t}{,} \end{aligned}$$

which can be interpreted as the displacement velocity of the iso-$\psi$ lines, or *isopleths* associated to $\psi$, *e*.*g*., the isotherms if $\psi$ is the temperature. This approach, hereafter termed the “gradient method”, received a widespread attention, partly because of its ease of implementation and the availability of software packages^7^. It has been identified as a powerful tool for projecting future species distributions and determining those at risk^6,8^. Such information allows to target conservation actions like (re)locating protected areas where the populations of interest would find suitable conditions and/or to which they would be able to shift in a projected climate scenario. It also allows to identify species that are not capable of following the pace of climate change, and so could benefit from interventions like assisted migration. ^9–13^. Alternatively, regions displaying low climate change velocity may be sought as offering more stable conditions, and therefore be more favourable for conservation^14^.

Such conservation policy planning relies on the expectation that the gradient-based velocity of climate change predicts well the species range shift in both magnitude and direction. However, a direct comparison of species range shifting velocity with the velocity of climate change calculated using the gradient method shows very scattered results ^15^, or even fails to establish a correspondence between the respective velocities of the range shift and of climate^16^, except in the case of marine species^17^. Furthermore, most work to date has focused on the magnitude of the shifting velocities, without explicitly considering longitude, latitude and depth/altitude separately (*e*.*g*.^18^). Most focused exclusively on shifts in latitude^16,19,20^, and none to date considered longitude. In contrast, ecological records show that species ranges shift in latitude, longitude, and in elevation/depth, the latter playing a key role in adaptation to increasing temperatures^21^, thus deserving specific assessment. The three components of shifting velocities must therefore be taken into account explicitly^15,22,23^.

The limitated agreement between the velocity of climate change and species range shift is to some extent related to the complexity of biological behavior during displacements, including the interaction between species (e.g., in the case of whales^24^), as well as topography^8^ and other ecological barriers^25^. Obstacles also cause delays that result in a time lag between the climate change and the species range change, especially for taxa with low individual mobility such as vegetal species^26^.

**Fig. 1:**
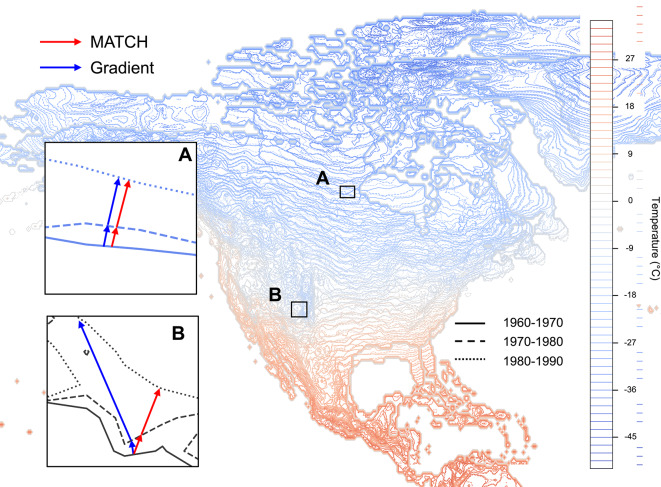
Displacement of the winter isotherms (DJF) between the decades 1960–1970, 1970–1980 and 1980–1990. The insets show the respective trajectories inferred using the Gradient and MATCH approaches in the case where the isotherms are locally parallel (A) or not (B) to each other. This figure was generated using matplotlib 3.7.0, on python 3.10.12 on Anaconda Jupyter Notebook 6.5.2, https://pypi.org/project/matplotlib/3.7.0/.

Besides ecological issues, the gradient approach has intrinsic limitations. It assumes that the isopleths shift down the gradient of the underlying field, i.e. that their displacement is locally perpendicular to the isopleth. This assumption is justified for parallel isopleths undergoing a uniform climate change at each latitude^27^ (Fig. 1A). However, in general, the isotherms will not face a homogeneous surface on Earth due to the topography and the land-ocean difference, which will deform them and strongly influence their path. In this case, the above assumption is invalid, as exemplified in Fig. 1B.

Furthermore, Eq. (1) causes the velocity to locally blow up towards infinity at grid points where the gradient vanishes. This artifact is evidenced in Burrows et al.^6^ where the histogram of the velocities has very long tails, up to highly unrealistic magnitudes. For this reason, the same authors suggested that the gradient approach should only be applied to shifts smaller than the grid resolution to limit the risk of unrealistic jumps^8^.

Facing these limitations of the gradient approach (labelled as *gridded approach* in their work) to track the range shift of marine species, Pinsky et al.^20^ developed an alternative approach conceptually similar to the climate analogues^28^, sometimes also termed climate twins^29,30^. The latter consists in identifying locations displaying a present climate similar to the projected climate of a location of interest. With this in mind, Pinsky et al.^20^ define the shift of the distribution range of a given species by identifying where analogous conditions for the considered species are found in the neighborhood of their original location. While the recent publication of the Climetrics *R* package^31^ facilitates its application, this method is highly dependent on the chosen aggregation criteria in the definition of the similarity of climate conditions^32^. Chen et al.^33^ developed yet another approach, in which they determined the displacement of individuals to the nearest location where the conditions are similar, rather than along the gradient, obtaining remarkable agreement between the species shift and the climate change velocity. However, this approach relies heavily on the assumption that analogous conditions can be found in a reasonably close neighborhood.

In order to address the limitations of the gradient approach from a more fundamental point of view, a local aggregation of the estimated velocities based on stochastic processes was proposed^34,35^. The Monte-cArlo iTerative Convergence metHod (MATCH) approach further builds in this direction. MATCH differs from the gradient method in the assumptions introduced to define the direction of the climate velocity shift. While as described above, the gradient approach assumes the shift to occur down the gradient, MATCH locally determines the direction of the velocity in a way that minimises the shear of the velocity field, i.e. that maximises its regularity. Unless isotherms are straight and move parallel to each other as it is the case in Fig. 1A, this constraint results in predicting trajectories substantially different from those obtained with the gradient-based method, as exemplified in Figure 1B. From an algorithmic point of view^36^, MATCH iteratively tests local random deformations of the computational grid. These deformations are kept or rejected based on their ability to move the original isopleths closer to the final ones. Once the iterative algorithm has converged, the final displacement of each original grid vertex defines the velocity of climate change at that location for the climate variable under investigation. By providing a continuous velocity field with locally constrained displacements, this approach is more likely to prevent unrealistically fast velocities.

Here, we provide a first comparison of the respective relevance of the gradient and MATCH approaches to estimating the velocity of climate change in the context of species range shifts. Relying on long-term observational databases of birds and marine species, we calculate the displacement of the centroid of each species’ distribution area and compare it with the mean velocity of climate change computed over that area using either method. This comparison is performed on each coordinate: latitude, longitude, and elevation (resp. depth) for birds (resp. marine species).

## Results

**Fig. 2:**
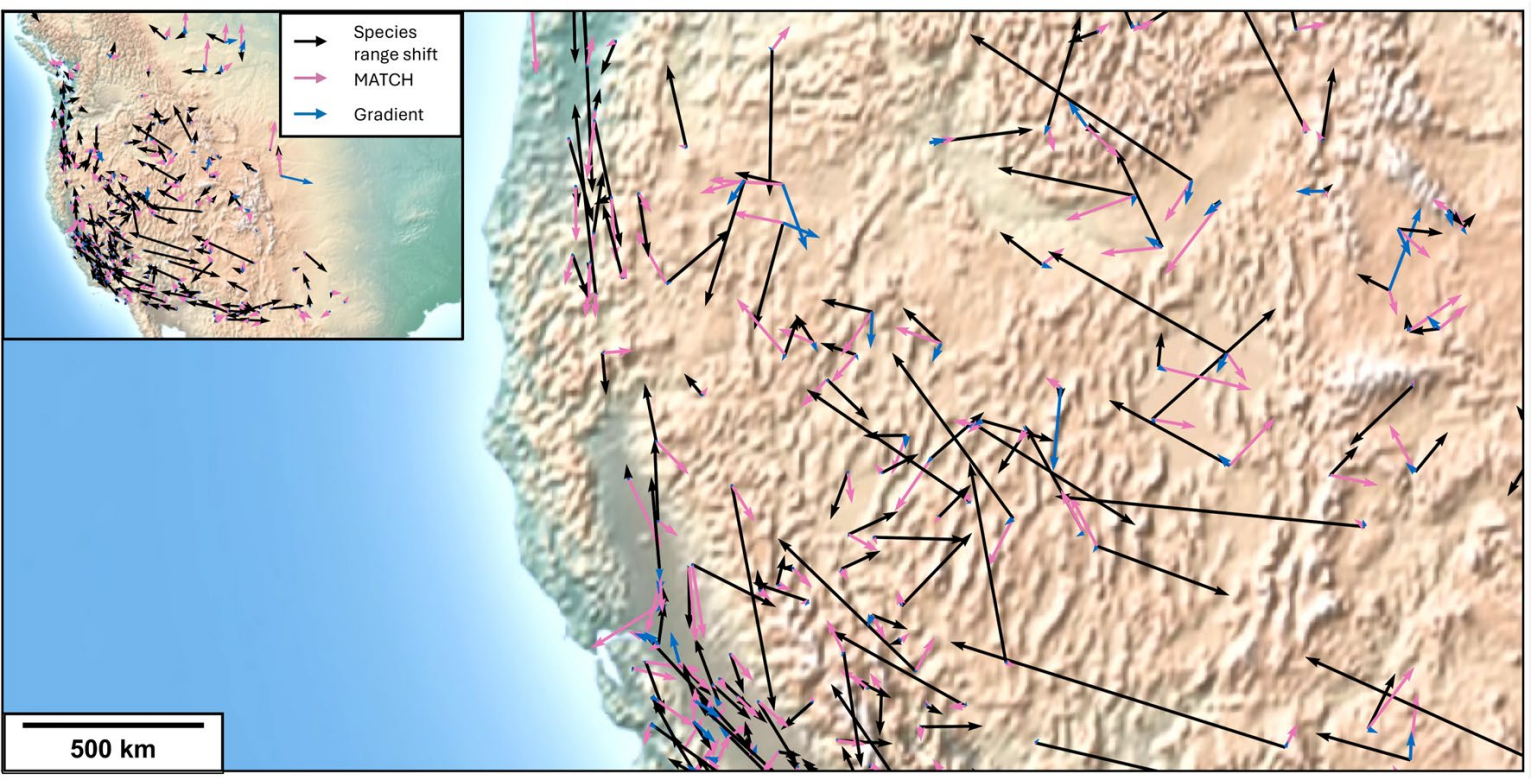
Velocity of climate change (represented by the surface air temperature) at the center of mass of bird species distribution ranges in the Western part of North America, between 1970–1980 and 1980–1990, calculated using both the MATCH and the gradient methods. This figure was generated using basemap 2.0.0 on python 3.10.12 on Anaconda Jupyter Notebook 6.5.2, https://pypi.org/project/basemap/.

### Birds, western North-America

We compare the observed range shifts of North-American birds and marine species with the velocity of climate change calculated using either the gradient-based or the MATCH method. We perform the comparison in latitude, longitude, as well as elevation (resp. depth for marine species), for decade-to-decade shifts, as well as the aggregation of all available decades and between the first and last decade of the considered period. All coefficient and slope values are found in Table S4 and Table S5.

Figure 2 shows the displacement of the observed distribution range centroids of the 200 bird species in western North America that met the inclusion criteria, compared with the displacement of climate in the same region, between the decades 1970–1980 and 1980–1990 (see Table S1). The climate displacement vector is calculated using either the gradient or MATCH methods. In the western part of the computational domain (longitude $< -100^\circ$), many blue arrows corresponding to the gradient method are missing. In the algorithm, these velocities have been set to zero since the very small local temperature gradients imply unrealistically large velocities (See Eq. 1). In contrast, the MATCH approach (red arrows) provides velocities across the whole domain and seems closer to the observed range shifts especially in terms of heading.

**Fig. 3:**
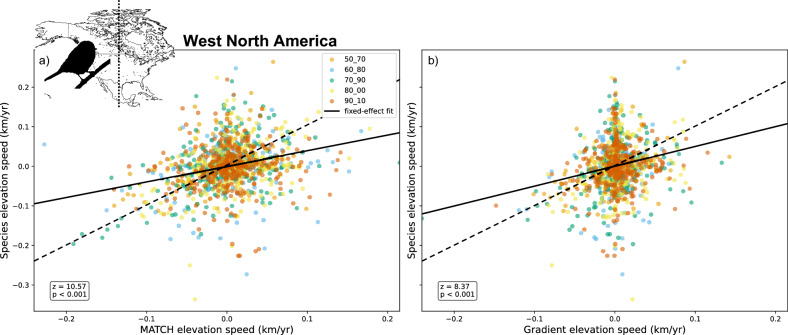
Comparison of species range shifts from the Audubon Christmas Bird Count in western North America (longitude $\le -100^\circ$) in the elevation direction, with climate shift calculated by the MATCH method (left panel) and by the gradient method (right panel) over all individual decades covering between 1950 and 2010. Each point on the graph corresponds to the shift of a species over a specific decade, see the legend for the correspondence between color and time frames. A linear mixed model has been used for each method; The *z* value and its corresponding *p* value are indicated. The doted line corresponds to the 1:1 line.

**Fig. 4:**
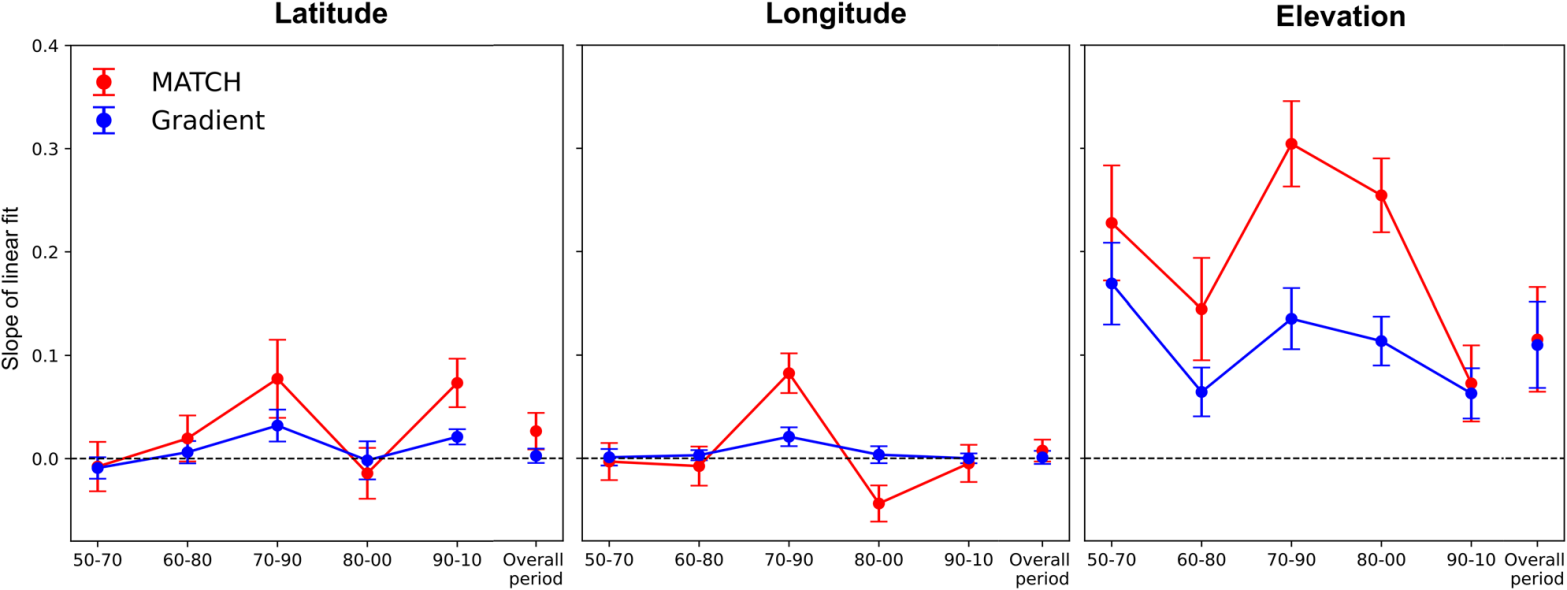
Evolution of the slope of the decade-to-decade linear regression of the bird species range shift velocity in Western North America, as a function of the climate velocity. Each coordinate is considered independently: (a) longitude, (b) latitude, (c) elevation. Confidence intervals correspond to the standard error of the regression slope. The first 5 points of each panel and the corresponding curves display the evolution over successive decades. The last one corresponds to the linear fit for the displacement over the whole period, i.e., from 1950–1960 to 2000–2010.

Figure 3 provides a more quantitative assessment of the above comparison by gathering the 1256 data points corresponding to 366 bird species aggregated over all decades from 1950–1960 to 2000–2010, in the part of North America located west of $-100^\circ$ longitude. To take into account the fact that the same species appears in several pairs of decades, we used a linear mixed model with the species as a random term. The MATCH and gradient methods (predictor) display significant effect on the species (response) only in the elevation direction with *z* values of 10.57 and 8.37 respectively. For latitude and longitude, no statistical significant effect where found. The absence of effect on the longitudinal and latitudinal velocities also translates in a very broad distribution of the heading differences between the species range shift and the velocity of climate change calculated with both methods (Fig. S2). This width is further enhanced by the contribution of the shifts with low velocity magnitude, where the uncertainty in the heading is very high; up to an undefined value for zero velocity.

For the decade-by-decade analysis, a linear regression is performed as the variable are linearly independent. Figure 4 displays the temporal evolution of the slope of the linear fits, together with the confidence interval of this slope. Both the slope and the correlations along all axes (see also Table S1) feature a peak in the 1980’s, which is known as a period of fast climate variation over North America^37^. Only elevation shows a significant correlation between range shifts and climate velocity for each decade-to-decade interval throughout the investigated period.

As for the individual decades, the overall shifts over the whole period under study (1950–1960 to 2000–2010) (Fig. 4, rightmost point of each curve and Figure S4) display significant correlations for the elevation, and for the latitude in the case of using MATCH method. Again, no significant correlation is observed on the longitude except for the MATCH method in the 1980’s. The overall lower correlation for the displacement over the whole period, as compared with decade-per-decade calculations, is related to the fact that the trajectory of species range shift is far from straight. We also note that the species shifting velocity along either axis is much slower than the climate velocity, especially when the latter is calculated with the gradient method (Fig. 3, Fig. S4, and Table S1).

###  Birds, eastern North-America

Correlations are much weaker in the eastern part of North America (longitude $> -100^\circ$), where bird range shifts are expected to be more dependent on precipitation than on temperature^38^ (Table S2). This highlights the need to adequately define the relevant climate variable(s) and statistical aggregates when assessing species range shift with regard to future climatic change. Furthermore, a similar analysis performed for the whole North America shows no clear trend either (Fig. S5), as can be expected for the aggregation of two samples with different behaviors.

###  Marine species

Table S3 provides a similar analysis for the 66 marine species in the North East Atlantic that met the inclusion criteria during the following periods: 1984–1994 to 1994–2004 and 1994–2004 to 2004–2014. As with birds, the MATCH method is much more closely related to the observed species’ latitudinal and depth range shifts than the gradient-based method. Correlation coefficients with the depth shift over the entire period (labelled “84–04” in Table S3) are respectively $r = 0.11\ (p = 0.41)$ for the gradient with a $slope = 0.09\pm 0.10$ and $r = 0.2{7}\ (p = 0.03)$ for MATCH with a $slope = 0.64\pm 0.29$, and amount to $r = 0.20\ (p = 0.05)$ with a $slope = 0.2 {8}\pm 0.1 {4}$ for the latitude in the case of MATCH. As with birds, longitudinal shifts displayed no significant correlations, and the overall displacements during the whole period of 1984–2014 show less correlation than decade-to-decade displacements, being positively correlated only to the climate shift over the elevation (depth) direction (Table S3).

The disparity between the regions becomes obvious when examining the histograms of the difference between the headings of the species range shift and of the climate change velocity (Fig. S2). In the case of North American birds, the mean is 10.17 $^\circ$ for MATCH and 13.52 $^\circ$ for the gradient method, both following a somewhat Gaussian shape. In contrast, the marine species exhibit a higher mean and standard deviation, as well as a tri-modal distribution. This is directly linked to the disparity of the correlation between the regions.

**Fig. 5:**
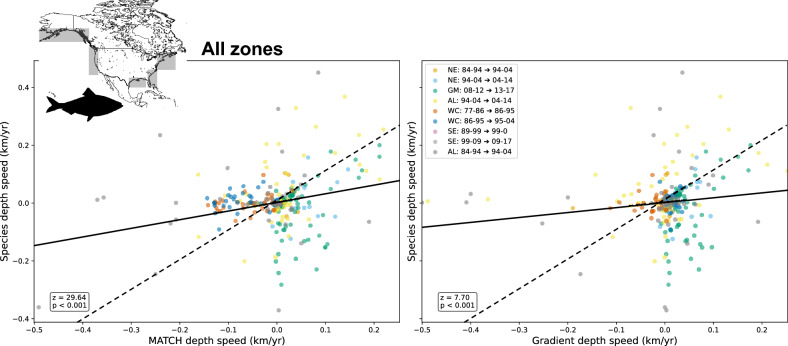
Comparison of all available decade-by-decade shifts in marine species ranges between 1977 and 2017, with climate shift calculated by MATCH (left panel) and Gradient (right panel) methods. Each point on the graph corresponds to the shift of a species over a specific decade and fishing region. Grey hatched zones represent the regions taken into account, See Fig. S1 for the detailed geographical extent. A linear mixed model has been used for either method; The *z* value and its corresponding *p* value are indicated. The doted line corresponds to the 1:1 line.

This link with depth and, to a lesser extent with latitude for MATCH, is also observed in the aggregated data analyzed using a mixed linear model for all fishing zones, as shown in the joint scatter plots (Fig. 5) and Table S6. Table S3 displays all corresponding fit slopes, correlations, and associated *p*-values for the fishing zones where marine species met the inclusion criteria (390 species in total).

## Discussion

Our analysis shows that correlations between species range shifts and the velocity of climate change are stronger on the elevation/depth direction. They are smaller but still remain significant on the aggregated decades and for several individual decades in the case of latitudinal shifts, and are rare for longitude. Furthermore, when present, the correlation is stronger and the slope of the linear fit is closer to one when considering climate velocities calculated with the MATCH method rather than with the gradient-based method. In all cases, however, the climate shifting velocity is faster than the species range shift.

Therefore, these results firstly indicate a higher relevance of the MATCH climate change velocity field as compared to the gradient-based one for analyzing range shifts of Western North-American birds as well as of marine species. This is especially the case in the latitudinal and elevation/depth directions as evidenced by the Pearson correlation coefficients and the slopes of the linear fits. Conversely, along the longitudes, climate velocity is generally not correlated with the observed longitudinal displacement of bird and marine species distribution ranges. This suggests that temperature has a minimal influence on the longitudinal range shifts.

The shift of western American birds is more correlated with the velocity of climate change calculated by either approach over the short term (decade to decade), than over a longer time interval. This may be due to the variability in both climate and range shift conditions over time as well as in space, which results in irregular trajectories making the interpretation of long-term shifts more difficult.

The period of 1970–1990 displays exceptional correlations and higher slope as compared to the rest of the study period, along all dimensions. This is likely related to the “1980s regime shift”^37^ (See the high rate of temperature change in Fig. S3). During that period, Western American birds have been facing a more abrupt change in the climatic conditions, leading to a higher sensitivity to temperature as compared to the remaining of the study period. This transient climate perturbation was strong enough to particularly influence the bird range shifts, up to the point where significant correlations are even observed across longitudes. This relationship is more pronounced in the case of the MATCH method.

Except for the peak in the 1980s, correlations (and to a lesser extent the associated slopes of the linear fits) in the latitudinal direction display an increase in the last decade and a small increasing trend along the decadal decomposition (Fig. 4 and Table S1). This trend may be related to the acceleration of climate warming in the last decades^39^. It may also be related to limitation of elevation shifts in the search for optimal temperatures. Moreover, elevations that are too high can lead to adverse conditions, such as decreasing atmospheric pressure and associated oxygen depletion^40^.

In contrast with Western North American birds, Eastern North American ones only show weak correlations with the elevational shift of temperature. This is consistent with the expected dominant influence of precipitation over temperature on the birds in this region.^38^.

Marine species display no consistent behavior between the considered fishing regions. The West and South-East Coast regions, as well as the Gulf of Mexico, do not show any clear correlation in any direction. This lack of correlation can be explained by the coastal orientation. As these regions are stretched along the coast, shifts can only occur in one dimension, parallel to the coast. Similarly, most shifts of species in the Gulf of Mexico face a continental barrier against latitudinal and longitudinal shifts. The absence of link between species range and climate shifts in these regions may also be due to the limited temperature increase they experience over the period of study.

In contrast, the two most northern marine regions in our dataset, namely, Alaska and the Northeast coast, experienced an increase in temperature by up to $14^{\circ }$C locally between 1984–1994 and 2004–2014. For the Alaska region, this is explained by the loss of sea ice up to a reduction of 15.9% per decade. This loss causes a decrease in albedo, which leads to an increase in temperature^41^. For the North-East coast a severe increase in the number of days with marine heatwaves has been recorded over the decades^42^. In relation with this strong and fast warming, these regions display high correlations and slope values closer to one for both methods. Again, the MATCH method provides stronger correlations and slope values closer to unity, especially for depth. This pronounced vertical motion is enhanced by a marked continental/coastal barrier for both regions. Therefore, species need to shift to deeper water to track their thermal niche, as it has already been shown^43^.

Even when the *p*-values indicate a high statistical significance, the correlation coefficients remain moderate and slopes stay below unity, especially in the case of the gradient-based method (Figs. 3, 5, Tables S1 and S3). This underlines the need to adequately choose (and potentially combine) climate parameters and their statistical aggregates (mean, extreme values, etc.), but also non-climatic parameters. As we are considering only a single parameter (temperature), we may underestimate the overall climate velocity^35^. In addition, we do not consider other non-climatic factors such as phenology adaptations, that are known to influence the range shifts of species^2^. In particular, Huang et al.^23^ and Bateman et al.^22^ both observed a strong longitudinal component in the shift of birds distribution ranges, although the aggregation methods in these two studies are slightly different from each other and from the present study. Furthermore, over land, dominant influences on species shift may include biological barriers^25^ such as topography and biome boundaries (which in turn may also shift in response to climate change^44^), but also large-scale intensive farming^45^.

Despite these limitations, our results definitively show that the MATCH approach is more relevant to the velocity of climate change than the gradient-based one. This may seem counter-intuitive from an ecological point of view. It seems natural to assume that in the absence of barriers, species or biomes would follow a shift along a route minimizing a generalized cost.

However, distance is only one component of travel costs. The travel time, the efforts required to overcome adverse winds, currents^46,47^, or obstacles such as topography, the availability of habitat or food, the large urban or intensively farmed areas^45^, or, in the case of marine species, the availability of depth ranges adapted to the life-cycle of the species of interest^6^, may also contribute and are not taken into account when using either of the method. Furthermore, in a non-stationary situation where curved isotherms deform with time, the local changes in the patterns in the temperature map impact trajectories. Pathways minimizing distance therefore have no reason to follow the gradient. (See Inset B of Fig. 1 and Fig. 1 of Gaponenko et al.^48^). An explicit simulation of these aspects is however beyond the scope of the present work.

Finally, and more importantly, even if the travel costs were to fully describe the shift costs for an individual (or a synergetic group like a colony or flock), the situation is more complex for populations covering a finite distribution range. Crowding effects come into play in this case. The high (low) population or group density and associated access to resources would locally increase (decrease) the relocation cost. As a consequence, shifts that result in more homogeneous population distributions, hence in more regular velocity fields as those provided by the MATCH method, may be favored. In contrast, the gradient method causes the velocity vectors to point locally towards hot spots (locally convergent velocity field), or from source regions (divergent velocity field). Range shifts following such convergent (resp. divergent) velocity fields would result in over- (under-) crowded regions. This crowding would induce an additional component to the cost function, due to the associated competition for resources (food, space...)^49^. Such crowding contribution to the cost of the range shift would translate into density-dependent regulation of the population in the target region and therefore tend to regularize (*i*.*e*., reduce the vorticity of) the species range shifting velocity field as the MATCH method does. Indeed, substantial anisotropies and deviations from a poleward shift have recently been observed in numerous species^50^, with no indication of strong constraints by topographic barriers. Such effects may explain the better correlations of the MATCH velocity of climate change with species range shifts, as compared with the gradient-based one.

## Conclusion

In this study, we have assessed two of these approaches, namely the gradient-based and the MATCH methods, against observational data of marine species and bird distribution ranges. As a general observation, regions and time periods with significant temperature increases have a better agreement with the MATCH method, leading to larger correlations and slope values closer to 1. This is particularly the case in the elevation/depth component and in some cases for the latitude. Nevertheless, the latitude and the longitude component of the velocity for marine species could not have been properly assessed, due to the elongated shape of the collecting region. Long-term shifts can be associated with tortuous trajectories that are likely difficult to match. As a consequence, short-term (decade-to-decade) shifts display stronger correlations than can be observed over several decades. This limitation, already observed in the case of the gradient approach and leading to focus on shifts of limited magnitude^8^, also seems to apply to MATCH.

The MATCH approach provides more significant correlations and slopes closer to one in both latitude and elevation/depth, compared to the gradient-based method. Our results therefore suggest that the MATCH approach may provide more ecologically relevant shifting climate velocity fields. However, further studies are needed to reach more robust conclusions. These include the consideration of more diverse taxa and regions worldwide. Furthermore, reducing distribution shifts to the corresponding centroid may be an oversimplification. In particular, the trailing edge of the distributions may be more sensitive to climate than the leading edge^51^. Finally, the consideration of multiple climate variables, either individually or in combination, may also help to better describe the impact of climate on species range shifts.

## Methods

### Datasets

#### Ecological observational data

Bird data have been obtained from the Audubon Society’s Christmas Bird Count (CBC)^52^. It provides decades-to century long human visual observations, including counting, of birds at the same locations each year at a fixed time frame in Winter across the whole North-American continent. However, we focused our analysis on the United States and Canada to warrant the continuity of the data series over the period 1950 – 2010. As observations are performed by volunteers, densely populated areas are likely over-represented in the database. This potential bias could not be corrected for and must be taken into account when interpreting the results.

To minimize biases, we applied a series of inclusion criteria for species to be taken into account in the study. Bird species were selected if they have been reported at least 5 times in each year of the initial and final decades of the time frame under investigation. Observation spots were included only if observations have been conducted each year during the same period of time, with at least two observations of the considered species. This is to limit the impact of identification errors.

Potential biases due to the boundaries of the study domain include both shifts beyond the observation region that would be overlooked by the analysis, and hard limits to shift (e.g., coastlines) that would bend or limit trajectories independently from the climate. We evaluated the impact of these side effects at the boundaries of the territory under study, by optionally excluding species with a range initially covering more than half of the area under investigation. The rationale for this criterion is that such wide territory is likely to cross these boundaries during the range shift. We however found that this limitation in the species range area does not affect the results as shown when comparing Figure 4 and Figure S5. Consequently, this inclusion criterion was eliminated. In order to avoid any potential spurious behaviours associated with insularity, the analysis was exclusively focused on connected territories. Specifically, the analysis of avian data excluded Hawaii. Typically 200 species are observed in each time period by taking into account 40 to 200 observations sites that followed the inclusion criteria.

For marine species, we relied on the NOAA Global Marine Data database^53^, as in Pinsky et al.^20^. The collection of data was conducted on a year-round basis by means of cameras installed on bottom trawls. The database covers 8 fishing regions along the US coasts, with dimensions in the range of 50,000 km$^2$ each (See Figure S1). They cover a total of 400,000 km$^2$, and gather 536,000 observations regarding 408 species. The inclusion criteria were similar to those described above for birds. However, as NOAA database does not define specific observation spots, criteria related to the latter are replaced by the requirement that a species should be observed at least 3 times per year to be included. The application of inclusion criteria restricted the database to five zones. We analyzed each zone separately, since the data were available over different time frames: Alaska (1984–2019), Gulf of Mexico (2008–2017), North-East coast (1984–2014, whereby fall and spring data are available separately, but were combined in our analysis), South-East Coast (1989–2017), and West Coast (1977–2004). The automation of the measurement ensures a temporally continuous sampling, providing binary presence-absence data^54^ for each year at each location.

#### Climate data

Surface air and sea surface temperature are considered in this study as as proxy for climate. The data were obtained from Copernicus Climate Change Service. For marine data, we focused on the annual mean of the sea surface temperature assembled from satellite data between 1977 and 2019, at a daily temporal resolution and $0.25^{\circ }$ x $0.25^{\circ }$ spatial resolution^55^. For birds, we used Winter (December, January, February, or DJF) screen-level surface air temperature over North America from the ERA5 reanalysis, offering a $0.5^{\circ }$ x $0.5^{\circ }$ spatial resolution with a hourly time step between 1901 and 2019.^56^ Due to year-to-year fluctuations (as well as seasonal fluctuations in the case of marine species) the climate data were averaged over 10-year periods. Wherever possible, we have carried out a decade-by-decade analysis. In all cases, we also investigated the overall range shift over the entire period for which data were available.

### Data processing

#### Displacement of distribution area

For each species the center of mass of each year’s distribution area is calculated as the geodesic mean position of all observations^57^. In the case of birds where the number of individuals observed at each spot is available, the average position is weighted by this number of individuals. The resulting mean positions for each year are then again geodesically averaged to provide the mean position of the species range over each decade. The offset between the mean position during the initial and final decades yields the shift vector. We divide it by the time interval between the initial and final decades to convert it into a velocity.

#### Calculation of the velocity of climate change

The climate shifting velocity according to both the gradient and the MATCH methods have been calculated as follows. First, the mean annual and DJF temperatures have been averaged over the considered decade (initial, and final) for each grid cell of the climate data. Based on these temperature fields, we calculated the vector fields of the shifting velocity between these two decades on each grid cell. This calculation was performed using either the MATCH algorithm^36^ as described in Gaponenko et al.^48^, or the gradient method. The resulting velocity at the centroid of the initial species distribution range was considered to be the climate velocity experienced by the species during its range shift.

We performed a separate analysis on each coordinate (latitude, longitude, and elevation/depth). Horizontal velocities of both climate and species range shift were separated into their latitudinal and longitudinal coordinates. Elevation and depth shifts were calculated from the ETOPO digital elevation model^58^, offering 2000 meters grid-spacing. Heading and velocity magnitude were also considered. While together they deliver the same information on the horizontal velocity as latitude and longitude, they often provide a more intuitive information on the shifts. However, for small magnitudes, the heading becomes increasingly noisy up to the point of being undefined when the magnitude approaches zero. This way of displaying the velocity vectors is therefore harder to interpret in the statistical analysis. Furthermore, angles are wrapped with a 360$^\circ$ period (Figure S2). We will therefore mainly favored analyses in terms of latitude and longitude. From the point of view of the user, both the MATCH and gradient approaches require a scalar field (the considered climate variable) as an input, and output a vector field describing the velocity at each location. Therefore, the workflow is of similar complexity. Both are available as open source software^7,36^, allowing users to apply it without specific programming skills. Despite its much higher algorithmic complexity and associated required computing time, MATCH can be run on a desktop computer within several hours of computing time for a typical $0.5^{\circ }$ x $0.5^{\circ }$ grid.

#### Statistical analysis

To assess the ecological relevance of the MATCH method we computed the Pearson correlation coefficient *r* and the slope of the corresponding linear fit as well as the associated confidence interval (where outliers have been removed using a threshold value of 1000  km/yr for each component of the studyong velocity). In order to avoid biases related to the occurrence of the same species in successive decade pairs, we used a linear mixed model with species as a random term in the case of the aggregated decade analysis. The analyses were performed using the pearsonr, linegress library from scipy.stats 1.11.4 and the mixedlm library from statsmodels on python 3.10.12 on Anaconda Jupyter Notebook 6.5.2^59^. The figures were generated using the code provided in the supplementary material, using Anaconda Jupyter Notebook 6.5.2, using matplotlib 3.7.0 and numpy 1.23.5.

#### Sensitivity analysis

The recent North American climate history can be divided into two distinct regions^38^, as illustrated in Figure S3, which displays the decade-to-decade evolution of temperatures over North-America. In the West (longitude $\le -100^\circ )$, the climate is more contrasted, evolves faster, and temperature is expected to be the key factor for defining optimal ranges for birds. In contrast, in the East, the climate conditions, especially temperatures, are more homogeneous in both space and time, and precipitation tend s to be the climatic limiting factor for birds development. Moreover, primary producers have been shown to exhibit greater efficiency, a factor which has been demonstrated to result in a reduction of environmental stress and the concomitant necessity for species shift^38^. In order to perform our statistical assessment over consistent behaviors, we performed the analysis independently on two areas separated by the $-100^\circ$ longitude meridian. Beyond correlations of the species range shifts and the climate velocity on each dimension (latitude, longitude, and altitude/depth), we investigated possible crossed influence between these axes. For this purpose, we used a multilinear model using the latitudinal, longitudinal and elevation velocities as independent variables, as an alternative to the linear fits. All crossed terms of the multiple linear model had negligible and/or non-significant correlations. Thus, we focused the discussion on single-parameter linear fits on each dimension.

## Supplementary Information

Supplementary Information 1.

Supplementary Information 2.

## Data Availability

Ecological observational datasets used in the present study are available from NOAA^53^ for marine species, respectively on demand to the National Audubon Society^52^ for birds. Climate data were obtained from Copernicus (https://cds.climate.copernicus.eu). The processed data used to generate the figures together with the code used to generate it are available in the supplementary material. The Python code used to calculate the MATCH velocities is available in the Yareta repository of the University of Geneva^60^.
