# Correction: Comparing ecological relevance of climate velocity indices

**DOI:** 10.1038/s41598-026-51671-z

**Published:** 2026-06-05

**Authors:** Laure Moinat, Iaroslav Gaponenko, Stéphane Goyette, Jérôme Kasparian

**Affiliations:** 1 Institute for Environmental Sciences, University of Geneva, Bd Carl Vogt 66, 1211 Geneva 4, Switzerland; 2 Group of Applied Physics, University of Geneva, Rue de l’Ecole de médecine 20, 1211 Geneva 4, Switzerland; 3 DQMP, University of Geneva, Quai Ansermet 24, 1211 Geneva 4, Switzerland

Correction to: *Scientific Reports* 10.1038/s41598-025-32377-0, published online 13 February 2026.

The original version of this Article contained an error in Figure 1, where the image text rendered as symbols.

In addition, the legend for Figure 1 was inadvertently published with correction comments.

Furthermore, there was an error in Figure 4, where the error bars were omitted from the image.

Finally, the legend for Figure 4 was inadvertently published with correction comments.

The original Figures 1 and 4 and accompanying legends appear below.

**Fig. 1:**
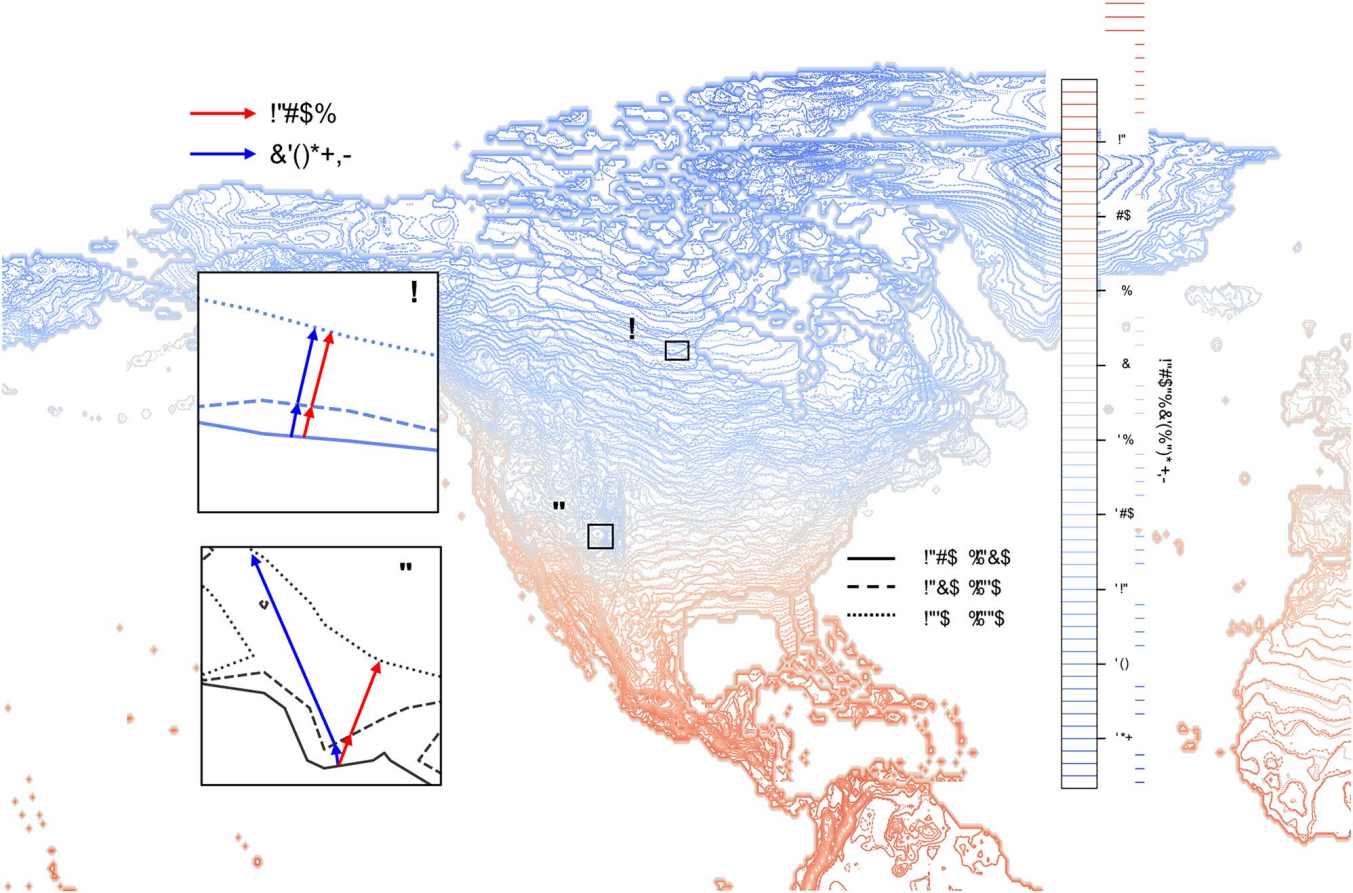
Displacement of the winter isotherms (DJF) between the decades 1960–1970, 1970–1980 and 1980–1990. The insets show the respective trajectories inferred using the Gradient and MATCH approaches in the case where the isotherms are locally parallel (A) or not (B) to each other. This figure was generated using matplotlib 3.7.0, on python 3.10.12 on Anaconda Jupyter Notebook 6.5.2. The figure is not displayed well, especially on the right side. I have uploaded a .pdf file named 'isotherme.pdf’ that should be used.

**Fig. 4:**
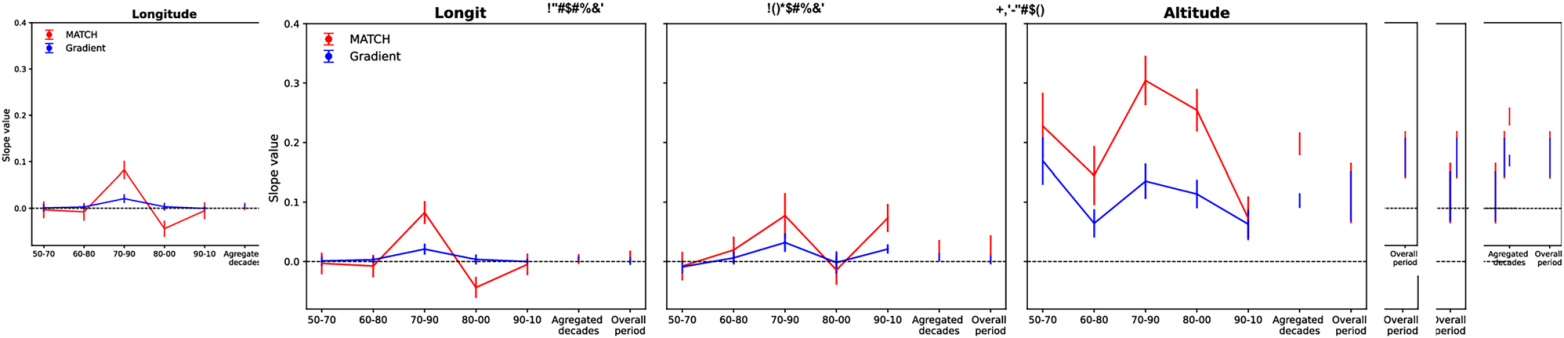
Evolution of the slope of the decade-to-decade linear regression of the bird species range shift velocity in Western North America, as a function of the climate velocity. Each coordinate is considered independently: (**a**) longitude, (**b**) latitude, (**c**) elevation. Confidence intervals correspond to the standard error of the regression slope. The first 5 points of each panel and the corresponding curves display the evolution over successive decades. The last one corresponds to the linear fit for the displacement over the whole period, i.e., from 1950–1960 to 2000–2010. The dots are not showing on the figure. I have uploaded the correct figure named 'Figure_4_MATCH_new.pdf’ .

The original Article has been corrected.

