# Supplementary material for: Comparing ecological relevance of climate velocity indices
Source: Sci Rep. 2026 Feb 13;16:8797. doi: 10.1038/s41598-025-32377-0 (PMC12982492; doi:10.1038/s41598-025-32377-0)
Supplement: Supplementary file 2 — Supplementary Information 2. [file 41598_2025_32377_MOESM2_ESM.pdf]

Supplementary material for : Comparing ecological relevance of climate velocity indices

Laure Moinat[1,2], Iaroslav Gaponenko[3], Stéphane Goyette[1,2], and Jérôme Kasparian[1,2,*]

[1]Institute for Environmental Sciences, University of Geneva, bd Carl Vogt 66, 1211 Geneva 4, Switzerland
[2]Group of Applied Physics, University of Geneva, Rue de l'Ecole de médecine 20, 1211 Geneva 4, Switzerland
[3]DQMP, University of Geneva, Quai Ansermet 24, 1211 Geneva 4, Switzerland
[*]jerome.kasparian@unige.ch

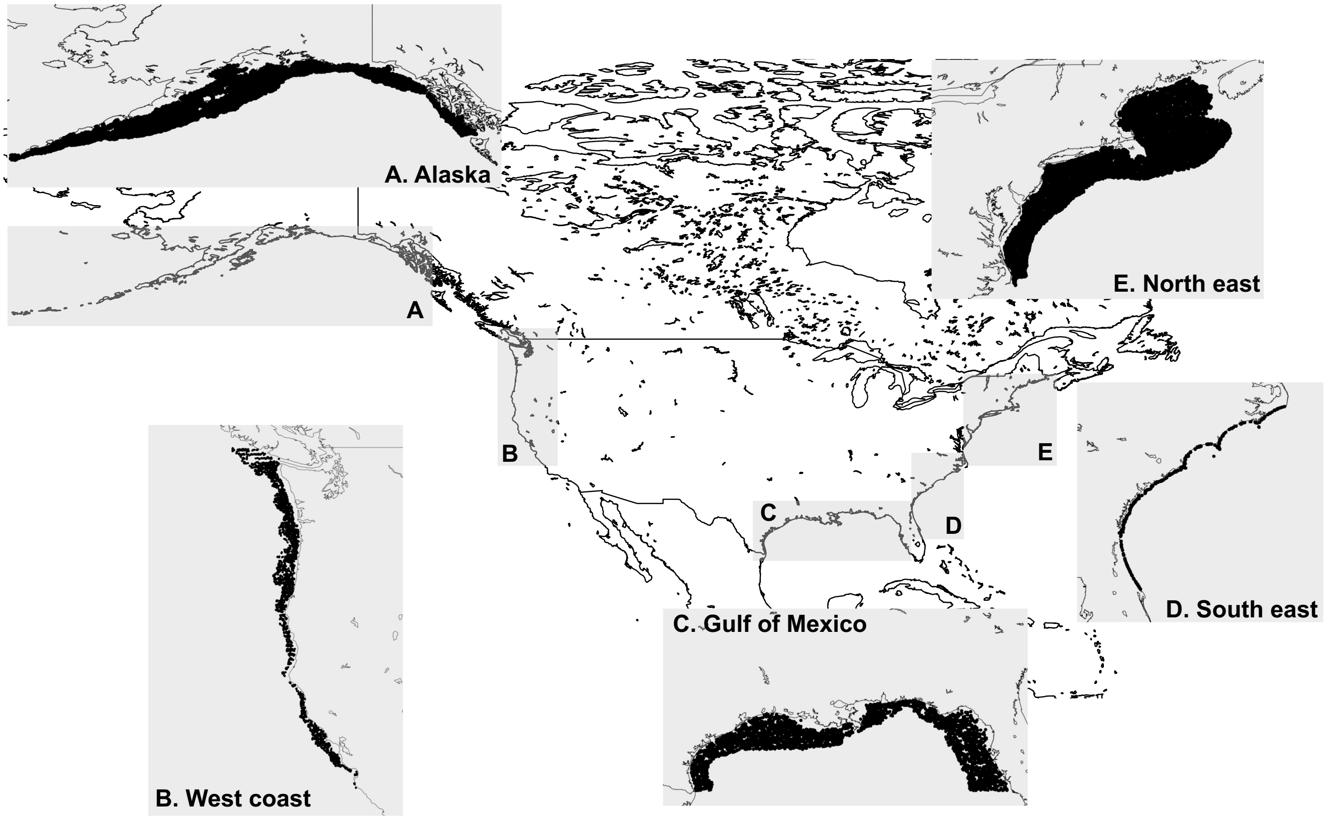

Figure S1. Location of the five zones from the NOAA Global Marine Database dataset included in the analysis. Each sample site appears as a black dot.This figure was generated using cartopy 0.25.0 on python 3.10.12 on Anaconda Jupyter Notebook 6.5.2, https://pypi.org/project/Cartopy/

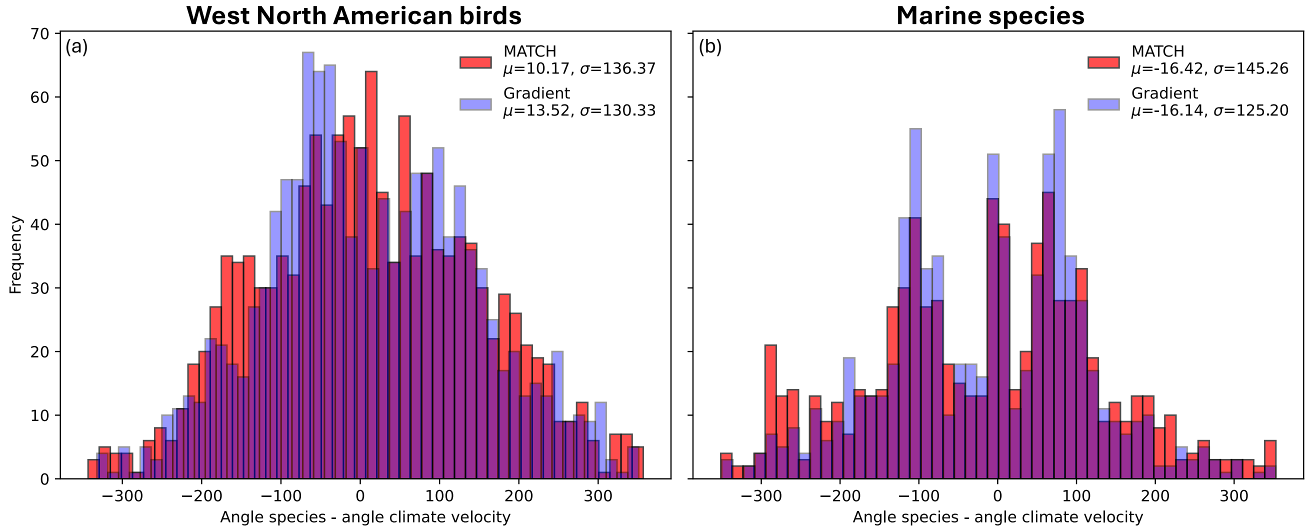

Figure S2. Histograms of the difference of the heading of the species range and climate velocity calculated using the MATCH and gradient based method in the case of (a) Western North-American birds, and (b) marine species. μ: mean, σ: standard deviation.

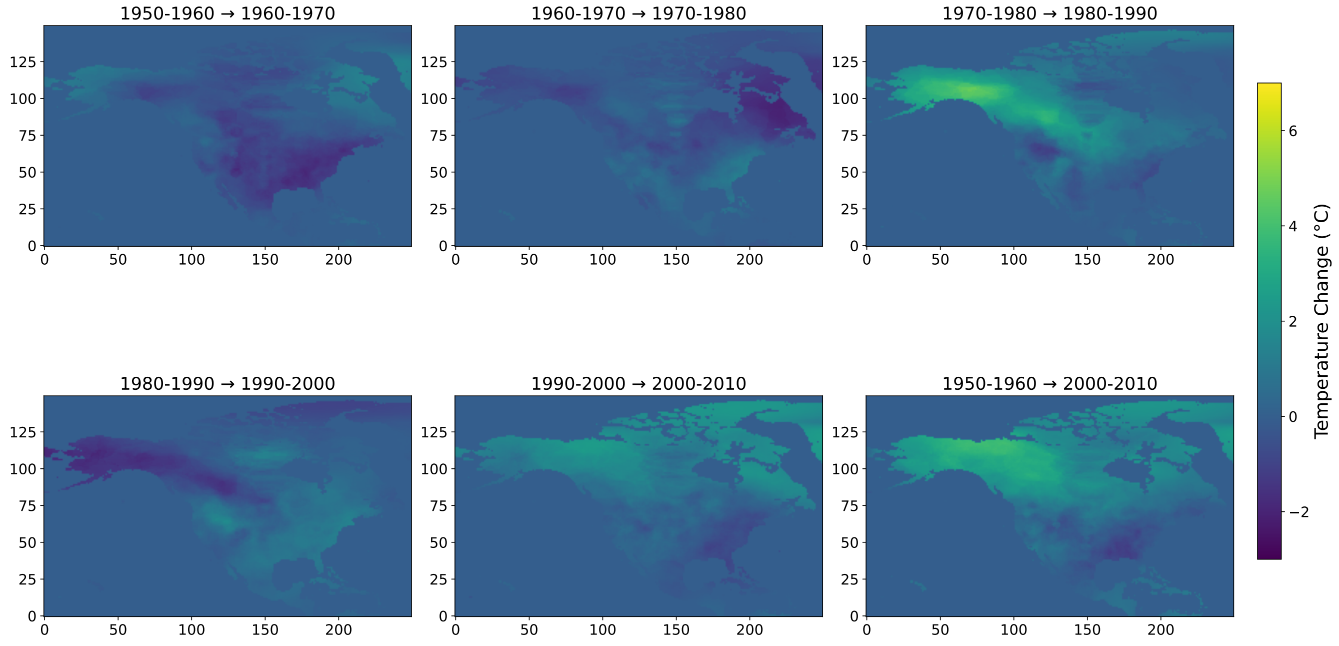

Figure S3. Changes in temperature over the North American continent during each time period. This figure was generated using cartopy 0.25.0 on python 3.10.12 on Anaconda Jupyter Notebook 6.5.2, https://pypi.org/project/Cartopy/

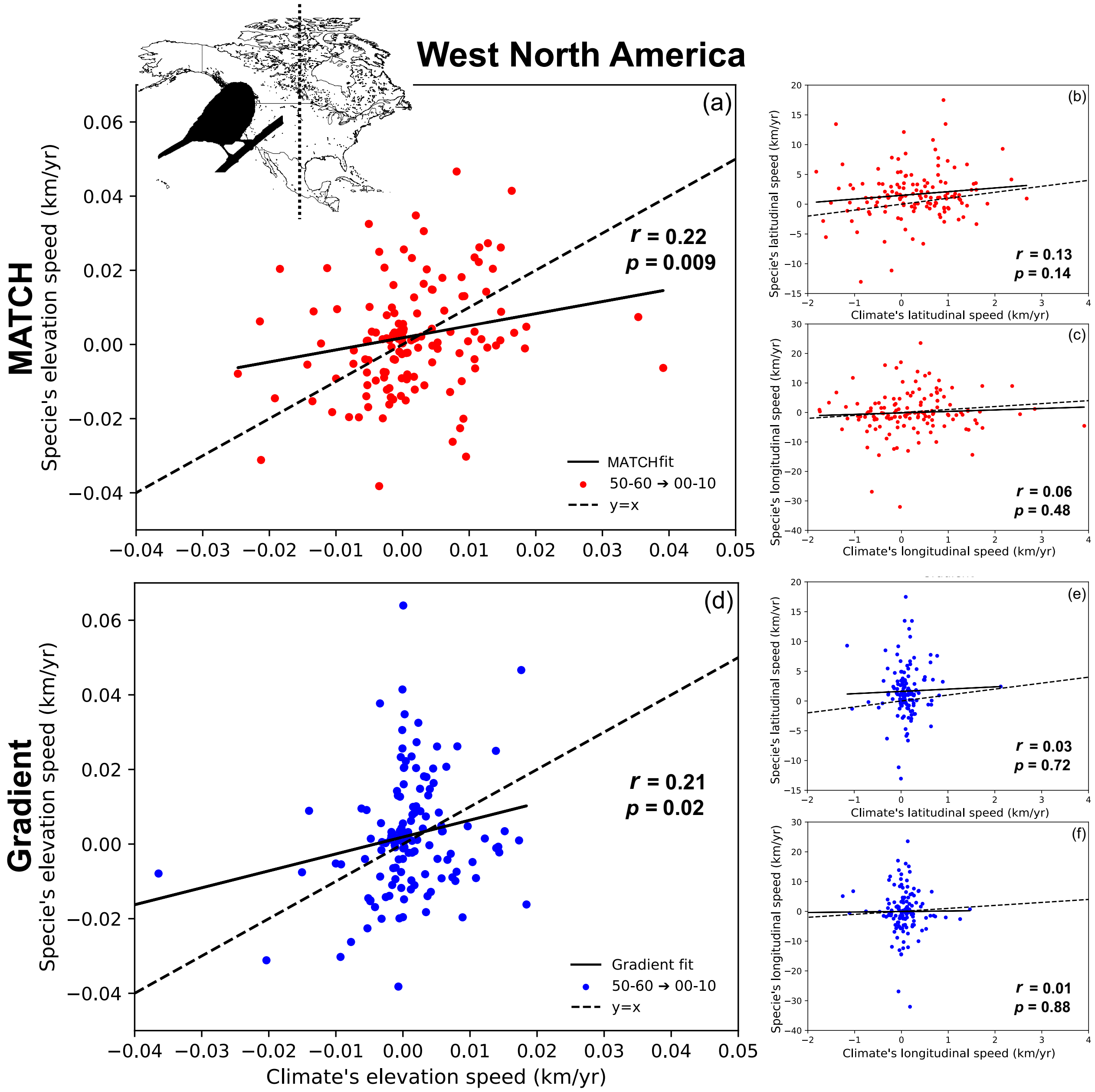

Figure S4. Comparison of the overall bird species range shift from the Audubon Christmas bird count in Western North America (longitude $\leq -100°$), between 1950–1960 and 2000–2010 with the climate shift calculated by (a,b,c) the MATCH method and (d,e,f) by the gradient method. Each point on the graph corresponds to the shift of a species over the considered time frame. Linear fits yield the correlation coefficients r and the associated statistical significances p between observed species range on each direction, and the corresponding climate shift calculated with either approach. (a,d): Elevation velocity (b,e): Latitudinal velocity, (c,f): Longitudinal velocity.

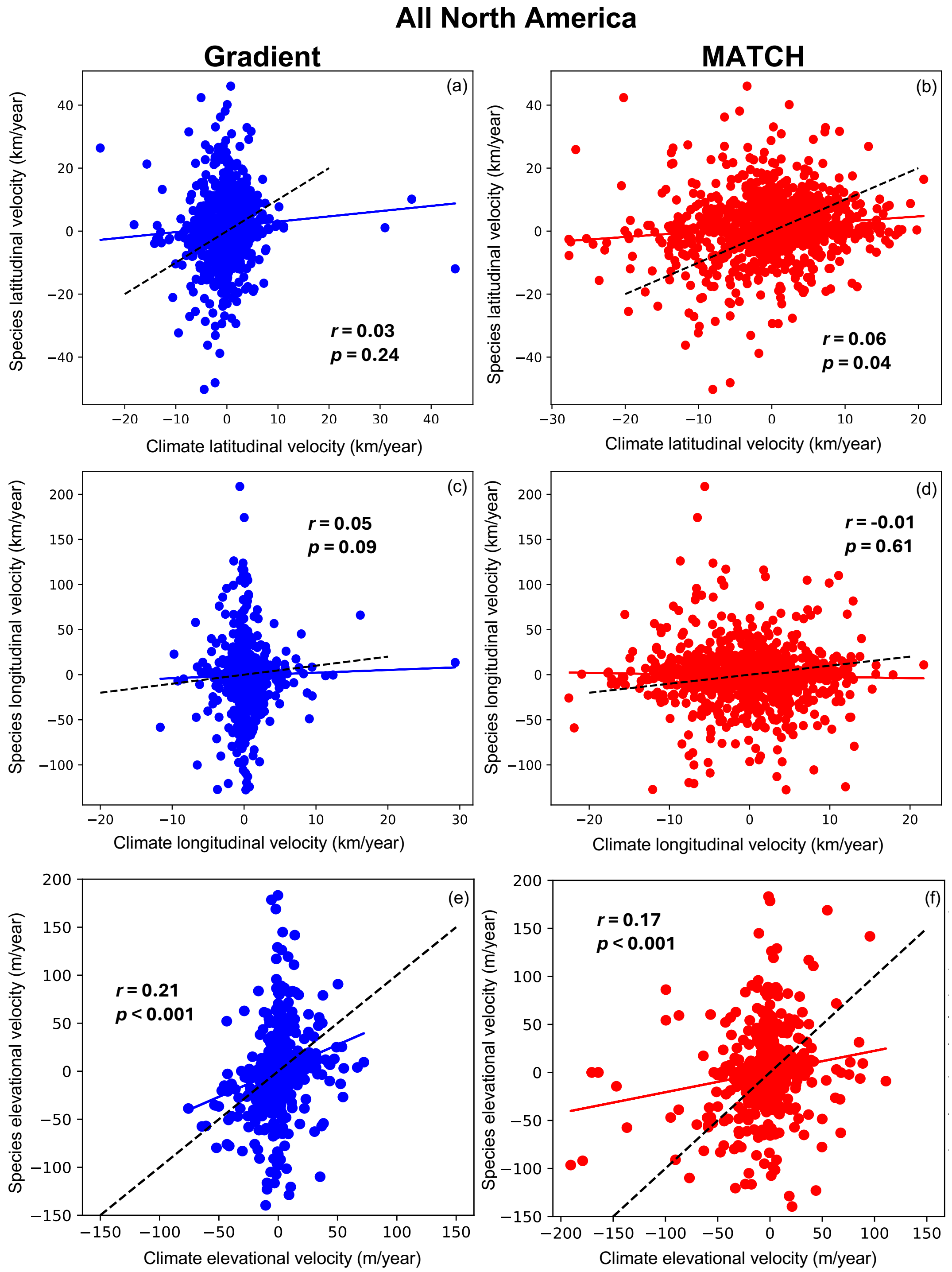

Figure S5. Comparison of the aggregated bird species range shift from the Audubon Christmas bird count for the entire North American continent, with the climate shift calculated (a,c,e) by the gradient method and (b,d,f) the MATCH method . Each dot on the graph corresponds to the shift of a species over the 1950–1960 to 2000–2010 time period. Linear fits yield the correlation coefficients r and the associated statistical significances p values between observed species range in each direction, and the corresponding climate shift calculated with either approach. (a,b): Latitudinal velocity,(c,d): Longitudinal velocity, (e,f): Elevation velocity.

		Latitude				Longitude				Elevation			
Time Period	Method	*r* value	*p*-value	Slope	Std Error	*r* value	*p*-value	Slope	Std Error	*r* value	*p*-value	Slope	Std Error
50-60 to 60-70	MATCH	-0.03	0.75	-0.01	0.02	-0.01	0.86	-0.01	0.02	0.33	<0.01	0.23	0.06
50-60 to 60-70	Gradient	-0.08	0.38	-0.01	0.01	0.01	0.90	0.01	0.01	0.34	<0.01	0.17	0.04
60-70 to 70-80	MATCH	0.06	0.38	0.02	0.02	-0.03	0.69	-0.01	0.02	0.19	<0.01	0.14	0.049
60-70 to 70-80	Gradient	0.04	0.57	0.01	0.011	0.04	0.54	0.01	0.005	0.18	<0.01	0.06	0.024
70-80 to 80-90	MATCH	0.12	0.04	0.08	0.04	0.25	<0.01	0.08	0.02	0.41	<0.01	0.30	0.04
70-80 to 80-90	Gradient	0.12	0.04	0.03	0.02	0.14	0.02	0.02	0.01	0.27	<0.01	0.14	0.03
80-90 to 90-00	MATCH	-0.03	0.56	-0.01	0.02	-0.14	<0.01	-0.04	0.02	0.37	<0.01	0.25	0.04
80-90 to 90-00	Gradient	-0.01	0.92	-0.01	0.02	0.02	0.67	0.00	0.01	0.26	<0.01	0.11	0.02
90-00 to 00-10	MATCH	0.16	<0.01	0.07	0.02	-0.01	0.78	-0.00	0.02	0.10	0.05	0.07	0.04
90-00 to 00-10	Gradient	0.15	<0.01	0.02	0.01	-0.00	1.00	-0.00	0.005	0.14	<0.01	0.06	0.02
50-60 to 00-10	MATCH	0.13	0.14	0.03	0.02	0.06	0.48	0.00	0.01	0.22	<0.01	0.10	0.05
50-60 to 00-10	Gradient	0.03	0.72	0.00	0.01	0.01	0.88	0.01	0.00	0.21	0.02	0.11	0.04

Table S1. Statistical analysis of birds in the western part of the North American continent.

		Latitude				Longitude				Elevation			
Time Period	Method	r value	p-value	Slope	Std Error	r value	p-value	Slope	Std Error	r value	p-value	Slope	Std Error
50-60 to 60-70	MATCH	-0.01	0.87	-0.01	0.05	0.01	0.88	0.01	0.03	0.13	0.09	0.07	0.04
50-60 to 60-70	Gradient	-0.03	0.69	-0.02	0.05	0.09	0.26	0.02	0.01	0.16	0.04	0.08	0.04
60-70 to 70-80	Match	0.05	0.42	0.02	0.03	0.01	0.86	0.00	0.02	0.13	0.05	0.05	0.02
60-70 to 70-80	Gradient	0.05	0.41	0.02	0.02	0.07	0.31	0.01	0.01	0.12	0.08	0.04	0.02
70-80 to 80-90	MATCH	-0.03	0.65	-0.19	0.10	0.01	0.00	0.01	0.10	0.01	0.00	0.01	0.10
70-80 to 80-90	Gradient	-0.06	0.31	-0.15	0.06	0.11	0.01	0.11	0.06	0.11	0.01	0.11	0.06
80-90 to 90-00	MATCH	-0.06	0.31	-0.03	0.57	0.03	0.62	0.03	0.57	0.03	0.62	0.03	0.57
80-90 to 90-00	Gradient	-0.07	0.22	0.11	0.39	0.05	0.06	0.05	0.39	0.05	0.06	0.05	0.39
90-00 to 00-10	MATCH	-0.13	0.02	0.04	0.49	-0.01	0.87	-0.01	0.48	-0.01	0.87	-0.01	0.48
90-00 to 00-10	Gradient	-0.08	0.12	-0.10	0.27	0.06	0.06	0.06	0.27	0.06	0.06	0.06	0.27
50-60 to 00-10	MATCH	-0.10	0.19	-0.01	0.76	0.05	0.54	0.05	0.76	0.05	0.54	0.05	0.78
50-60 to 00-10	Gradient	-0.02	0.77	0.04	0.16	0.18	0.02	0.18	0.16	0.18	0.02	0.18	0.16

Table S2. Statistical analysis for the eastern part of the North American continent for the birds

Region	Variable	Time	Method	*r* coefficient	*p*-value	Slope	Std error
Alaska	Elevation	94 -04	MATCH	0.51	<0.01	0.77	0.15
	Elevation	94 -04	Gradient	0.26	0.02	0.28	0.12
	Latitude	94-04	MATCH	-0.11	0.34	-0.11	0.12
	Latitude	83-19	MATCH	0.26	0.08	0.40	0.22
	Latitude	84-94	MATCH	0.18	0.25	0.12	0.11
	Latitude	94-04	Gradient	-0.02	0.88	-0.02	0.11
	Latitude	83-19	Gradient	0.08	0.59	0.08	0.14
	Latitude	84-94	Gradient	0.11	0.48	0.00	0.00
	Longitude	83-19	MATCH	0.37	0.01	0.68	0.26
	Longitude	94-04	MATCH	-0.14	0.23	-0.64	0.53
	Longitude	84-94	MATCH	-0.49	<0.01	-2.15	0.60
	Longitude	83-19	Gradient	0.18	0.23	0.00	0.00
	Longitude	94-04	Gradient	-0.11	0.32	-0.57	0.57
	Longitude	84-94	Gradient	0.04	0.80	0.00	0.01
South East	Elevation	99-19	MATCH	-0.40	<0.01	-0.71	0.20
	Elevation	89-09	MATCH	0.09	0.46	0.07	0.01
	Elevation	99-17	MATCH	0.01	0.91	0.02	0.18
	Elevation	99-17	Gradient	-0.00	0.98	-0.02	0.87
	Elevation	89-09	Gradient	0.04	0.73	0.11	0.31
	Elevation	99-19	Gradient	-0.30	<0.01	-2.22	0.88
	Latitude	89-17	MATCH	-0.25	0.04	-0.15	0.07
	Latitude	99-19	MATCH	-0.05	0.66	-0.06	0.13
	Latitude	89-09	MATCH	0.14	0.26	0.31	0.28
	Latitude	89-17	Gradient	-0.34	<0.01	-2.78	0.86
	Latitude	89-09	Gradient	-0.28	0.02	-1.74	0.72
	Latitude	99-19	Gradient	-0.08	0.50	-1.51	2.21
	Longitude	89-17	MATCH	-0.25	0.04	-0.15	0.07
	Longitude	89-09	MATCH	0.15	0.23	0.24	0.20
	Longitude	99-19	MATCH	0.12	0.32	0.11	0.11
	Longitude	89-17	Gradient	-0.34	<0.01	-2.78	0.86
	Longitude	99-19	Gradient	-0.03	0.78	-0.75	2.7
	Longitude	89-09	Gradient	-0.04	0.73	-0.75	2.16
North East	Elevation	84-14	MATCH	0.40	<0.01	0.42	0.10
	Elevation	84-04	MATCH	0.27	0.03	0.64	0.29
	Elevation	94-14	MATCH	0.35	<0.01	0.38	0.13
	Elevation	84-14	Gradient	0.38	<0.01	0.70	0.17
	Elevation	84-04	Gradient	0.11	0.41	0.09	0.10
	Elevation	94-14	Gradient	0.42	<0.01	0.67	0.18
	Latitude	84-14	MATCH	-0.25	0.01	-0.28	0.11
	Latitude	94-14	MATCH	0.24	0.15	0.27	0.12
	Latitude	84-04	MATCH	0.20	0.05	0.28	0.14
	Latitude	84-94	Gradient	0.11	0.29	0.00	0.00
	Latitude	84-14	Gradient	-0.08	0.44	-0.13	0.17
	Latitude	94-04	Gradient	-0.07	0.50	-0.05	0.08
	Longitude	84-94	MATCH	0.17	0.11	0.27	0.17
	Longitude	84-14	MATCH	-0.13	0.22	-0.21	0.17
	Longitude	94-04	MATCH	0.19	0.06	0.25	0.13
	Longitude	84-14	Gradient	-0.04	0.70	-0.07	0.18
	Longitude	94-04	Gradient	-0.07	0.49	-0.11	0.15
	Longitude	84-94	Gradient	-0.11	0.30	-0.00	0.00
West Coast	Elevation	77-95	MATCH	0.25	0.09	0.13	0.07
	Elevation	77-04	MATCH	0.25	0.08	0.13	0.07
	Elevation	86-04	MATCH	0.029	0.84	0.01	0.07
	Elevation	77-95	Gradient	0.06	0.70	0.06	0.14
	Elevation	77-04	Gradient	0.06	0.71	0.06	0.15
	Elevation	86-04	Gradient	0.13	0.38	0.26	0.29
	Latitude	77-95	MATCH	-0.22	0.13	-0.55	0.36
	Latitude	77-04	MATCH	0.16	0.30	0.39	0.37
	Latitude	86-04	MATCH	-0.02	0.86	-0.08	0.44
	Latitude	77-95	Gradient	-0.22	0.15	-1.21	0.83
	Latitude	77-04	Gradient	0.02	0.89	0.22	1.44
	Latitude	86-04	Gradient	-0.13	0.37	-1.27	1.41
	Longitude	77-95	MATCH	0.08	0.60	0.05	0.09
	Longitude	86-04	MATCH	-0.06	0.66	-0.03	0.07
	Longitude	77-04	MATCH	0.11	0.50	0.05	0.07
	Longitude	77-95	Gradient	-0.27	0.07	-0.42	0.22
	Longitude	86-04	Gradient	0.20	0.16	0.39	0.28
	Longitude	77-04	Gradient	-0.11	0.49	-0.17	0.2
Gulf of Mexico	Elevation	08-17	MATCH	0.23	<0.01	0.14	0.04
	Elevation	08-17	Gradient	0.20	<0.01	0.10	0.03
	Latitude	08-17	MATCH	0.10	0.16	0.10	0.07
	Latitude	08-17	Gradient	0.09	0.22	0.05	0.04
	Longitude	08-17	MATCH	-0.10	0.17	-0.04	0.03
	Longitude	08-17	Gradient	-0.32	<0.01	-0.04	0.03

Table S3. Statistical analysis of the marine species per region .

Direction	MATCH	Gradient
Elevation	r: 0.28<br>Coefficients: [-0.95, -11.86, 0.39]<br>y-intercept: -161.74<br>p: [0.51, 0.75, <0.001]	r: 0.23<br>Coefficients: [-0.47, 27.12, 0.50]<br>y-intercept: -90.57<br>p: [0.74, 0.71, <0.001]
Latitude	r: 0.08<br>Coefficients: [3.77 x 10^{-3}, 1.22 x 10^{-1}, -6.26 x 10^{-5}]<br>y-intercept: 0.71<br>p: [0.12, 0.06, 0.33]	r: 0.088<br>Coefficients: [0.00, 0.15, -0.00]<br>y-intercept: 0.70<br>p: [0.14, 0.24, 0.01]
Longitude	r: 0.02<br>Coefficients: [-2.23 x 10^{-3}, -3.68 x 10^{-2}, 1.47 x 10^{-5}]<br>y-intercept: -0.43<br>p: [0.56, 0.72, 0.88]	r: 0.08<br>Coefficients: [-0.00, -0.24, 0.00]<br>y-intercept: -0.43<br>p: [0.57, 0.22, 0.01]

Table S4. Multilinear regressions for each coordinate based on (x, y, z) from the MATCH and Gradient method for the aggregated decades for the West American birds.

Table S5. Linear mixed model result for the MATCH and Gradient method in the elevation, latitude and longitude direction for the birds in the Western North America.

Model and direction	Coefficient	z	p-value
MATCH elevation	0.017	10.571	<0.001
MATCH latitude	0.436	1.444	0.149
MATCH longitude	-0.478	-1.392	0.164
Gradient elevation	0.013	8.375	<0.001
Gradient latitude	0.271	0.897	0.370
Gradient longitude	0.577	1.687	0.092

Table S6. Linear mixed model result for the MATCH and Gradient method in the elevation, latitude and longitude direction for the marine species in the Western North America.

Model andd direction	Coefficient	z	p-value
MATCH depth	0.299	29.636	<0.001
MATCH latitude	0.030	6.760	<0.001
MATCH longitude	-0.167	-43.540	<0.001
Gradient depth	0.170	7.696	<0.001
Gradient latitude	0.006	0.927	0.354
Gradient longitude	-0.001	-0.037	0.971
